# Associations between medical students’ stress, academic burnout and moral courage efficacy

**DOI:** 10.1186/s40359-024-01787-6

**Published:** 2024-05-27

**Authors:** Galit Neufeld-Kroszynski, Keren Michael, Orit Karnieli-Miller

**Affiliations:** 1 Department of Medical Education, Faculty of Medical & Health Sciences, Tel Aviv University, Tel Aviv, 69778 Israel; 2 Department of Human Services, Max Stern Yezreel Valley College, Yezreel Valley, Israel

**Keywords:** Moral courage, Self-efficacy, Stress, Burnout, Medical students, Professionalism, Well-being, Patient safety

## Abstract

**Background:**

Medical students, especially during the clinical years, are often exposed to breaches of safety and professionalism. These contradict personal and professional values exposing them to moral distress and to the dilemma of whether and how to act. Acting requires moral courage, i.e., overcoming fear to maintain one’s core values and professional obligations. It includes speaking up and “doing the right thing” despite stressors and risks (e.g., humiliation). Acting morally courageously is difficult, and ways to enhance it are needed. Though moral courage efficacy, i.e., individuals’ belief in their capability to act morally, might play a significant role, there is little empirical research on the factors contributing to students’ moral courage efficacy. Therefore, this study examined the associations between perceived stress, academic burnout, and moral courage efficacy.

**Methods:**

A cross-sectional study among 239 medical students who completed self-reported questionnaires measuring perceived stress, academic burnout (‘exhaustion,’ ‘cynicism,’ ‘reduced professional efficacy’), and moral courage efficacy (toward others’ actions and toward self-actions). Data analysis via Pearson’s correlations, regression-based PROCESS macro, and independent *t*-tests for group differences.

**Results:**

The burnout dimension of ‘reduced professional efficacy’ mediated the association between perceived stress and moral courage efficacy toward others’ actions. The burnout dimensions ‘exhaustion’ and ‘reduced professional efficacy’ mediated the association between perceived stress and moral courage efficacy toward self-actions.

**Conclusions:**

The results emphasize the importance of promoting medical students’ well-being—in terms of stress and burnout—to enhance their moral courage efficacy. Medical education interventions should focus on improving medical students’ professional efficacy since it affects both their moral courage efficacy toward others and their self-actions. This can help create a safer and more appropriate medical culture.

## Introduction

In medical school, and especially during clinical years, medical students (MS) are often exposed to physicians’ inappropriate behaviors and various breaches of professionalism or safety [1–3]. These can include lack of respect or sensitivity toward patients and other healthcare staff, deliberate lies and deceptions, breaching confidentiality, inadequate hand hygiene, or breach of a sterile field [4, 5]. Furthermore, MS find themselves performing and/or participating in these inappropriate behaviors. For example, a study found that 80% of 3^rd–^4th year MS reported having done something they believed was unethical or having misled a patient [6]. Another study showed that 47.1–61.3% of females and 48.8–56.6% of male MS reported violating a patient’s dignity, participating in safety breaches, or examining/performing a procedure on a patient without valid consent, following a clinical teacher’s request, as a learning exercise [5]. These behaviors contradict professional values and MS’ own personal and moral values, exposing them to a dilemma in which they must choose if and how to act.

Taking action requires moral courage, i.e., taking an active stand or acting in the face of wrongdoing or moral injustice jeopardizing mental well-being [7–10]. Moral courage includes speaking up and “doing the right thing” despite risks, such as shame, retaliation, threat to reputation, or even loss of employment [8]. Moral courage is expressed in two main situations: when addressing others’ wrongdoing (e.g., identifying and disclosing a past/present medical error by colleagues/physicians); or when admitting one’s own wrongdoing (e.g., disclosing an error or lack of knowledge) [11]. 

Due to its “calling out” nature, acting on moral courage is difficult. A hierarchy and unsafe learning environment inhibits the ability for assertive expression of concern [12–14]. This leads to concerning findings indicating that only 38% of MS reported that they would approach someone performing an unsafe behavior [12], and about half claimed that they would report an error they had observed [15]. 

Various reasons were suggested to explain why MS, interns, residents, or nurses, hesitate to act in a morally courageous way, including difficulty questioning the decisions or actions of those with more authority [12], and fear of negative social consequences, such as being disgraced, excluded, attacked, punished, or poorly evaluated [13]. Other reasons were the wish to fit into the team [6] and being a young professional experiencing “lack of knowledge” or “unfamiliarity” with clinical subtleties [16]. 

Nevertheless, failing to act on moral courage might lead to negative consequences, including moral distress [17]. Moral distress is a psychological disequilibrium that occurs when knowing the ethically right course of action but not acting upon it [18]. Moral distress is a known phenomenon among MS [19], e.g., 90% of MS at a New York City medical school reported moral distress when carrying for older patients [20]. MS’ moral distress was associated with thoughts of dropping out of medical school, choosing a nonclinical specialty, and increased burnout [20]. 

These consequences of moral distress and challenges to acting in a morally courageous way require further exploration of MS’ moral courage in general and their moral courage efficacy specifically. Bandura coined the term self-efficacy, focused on one’s perception of how well s/he can execute the action required to deal successfully with future situations and to achieve desired outcomes [21]. Self-efficacy plays a significant role in human behavior since individuals are more likely to engage in activities they believe they can handle [21]. Therefore, self-efficacy regarding a particular skill is a major motivating factor in the acquisition, development, and application of that skill [22]. For example, individuals’ perception regarding their ability to deal positively with ethical issues [23], their beliefs that they can handle effectively what is required to achieve moral performance [24], and to practically act as moral agents [25], can become a key psychological determinant of moral motivation and action [26]. Due to self-efficacy’s importance there is a need to learn about moral courage efficacy, i.e., individuals’ belief in their ability to exhibit moral courage through sharing their concerns regarding others and their own wrongdoing. Moral courage efficacy was suggested as important to moral courage in the field of business [27], but not empirically explored in medicine. Thus, there is no known prevalence of moral courage efficacy toward others and toward one’s own wrongdoing in medicine in general and for MS in particular. Furthermore, the potential contributing factors to moral courage efficacy, such as stress and burnout, require further exploration.

### The associations between stress, burnout, and moral courage efficacy

Stress occurs when people view environmental demands as exceeding their ability to cope with them [28]. MS experience high levels of stress during their studies [29], due to excessive workload, time management difficulties, work–life balance conflicts, health concerns, and financial worries [30]. Studies show that high levels of stress were associated with decreased empathy [31], increased academic burnout, academic dishonesty, poor academic performance [32], and thoughts about dropping out of medical school [33]. As stress may impact one’s perceived efficacy [34], this study examined whether stress can inhibit individuals’ moral courage efficacy to address others’ and their own wrongdoing.

An aspect related to a poor mental state that may mediate the association between stress and MS’ moral courage efficacy is burnout. Burnout includes emotional exhaustion, cynicism toward one’s occupation value, and doubting performance ability [35]. Burnout is usually work-related and is common in the helping professions [60]. For students, this concept relates to academic burnout [36], which includes exhaustion due to study demands, a cynical and detached attitude to studying, and low/reduced professional efficacy, i.e. feeling incompetent as learners [37]. 

Burnout has various negative implications for MS’ well-being and professional development. Burnout is associated with psychiatric disorders and thoughts of dropping out of medical school [33]. Furthermore, MS’ burnout is associated with increased involvement in unprofessional behavior, eroding professional development, diminishing qualities such as honesty, integrity, altruism, and self-regulation [38], reducing empathy [31, 39] and unwillingness to provide care for the medically underserved [40]. Thus, burnout may also impact MS’ views on their responsibility and perceived ability to promote high-quality care and advocate for patients [41], possibly leading them to feel reluctant and incapable to act with moral courage [42]. Earlier studies exploring stress and its various outcomes, found that burnout, and specifically exhaustion, can become a crucial mediator for various harmful outcomes [43]. Although stress is impactful to creating discomfort, the decision and ability to intervene requires one’s own drive and power. When one is feeling stress, leading to burnout their depleted energy reserves and diminished sense of professional worth likely undermine their perceived power (due to exhaustion) or will (due to cynicism) to uphold professional ethical standards and intervene to advocate for patient care in challenging circumstances, such as the need to speak up in front of authority members. Furthermore, burnout may facilitate a cognitive distancing from professional values and responsibilities, allowing for moral disengagement and reducing the likelihood of morally courageous actions. This mediation role requires further exploration.

### Objectives

This study examined associations between perceived stress, academic burnout, and moral courage efficacy. In addition to the mere associations among the variables, it will be examined whether there is a mediation effect (perceived stress → academic burnout → moral courage efficacy) to gain more insight into possible mechanisms of the development of moral courage efficacy and of protective factors. Understanding these mechanisms has educational benefit for guiding interventions to enhance MS’ moral courage efficacy.

### Hypotheses

H1: Perceived stress and academic burnout dimensions will be negatively associated with moral courage efficacy dimensions.

H2: Perceived stress will be positively associated with academic burnout dimensions.

H3: Academic burnout dimensions will mediate the association between perceived stress and moral courage efficacy dimensions.

## Materials and methods

### Sample and procedure

A quantitative cross-sectional study among 239 MS. Most participants were female (60%), aged 29 or less (90%), and unmarried (75%). About two thirds (64.3%) were at the pre-clinical stage of medical school and about a third (35.7%) at the clinical stage. In December 2019, the research team approached MS through email and social media to participate in the study and complete an online questionnaire. This was a part of a national study focused on MS’ burnout [44]. The 239 participants were recruited by a convenience sampling. Data were collected online through Qualtrics platform, via anonymous self-reported questionnaires. The University Ethics Committee approved the study, and all participants signed an informed consent form.

### Measures

*Moral courage efficacy*—This 8-item instrument, developed for this study, is based on the literature on moral courage, professionalism, and speaking-up, including qualitative and quantitative studies [7, 13, 45–47], and discussions with MS and medical educators. The main developing team included a Ph.D. medical educator expert in communication in healthcare and professionalism; an M.D. psychiatrist expert in decision making, professionalism, and philosophy; a Ph.D. graduate who analyzed MS’ narratives focused on moral dilemmas and moral courage during professionalism breaches; and a Ph.D. candidate focused on assertiveness in medicine [14]. This allowed the identification of different types of situations MS face that may require moral courage.

As guided by instructions for measuring self-efficacy, which encourage using specific statements that relate to the specific situation and skill required [48], the instrument measures MS’ perception of their own ability, i.e., self-efficacy, to act based on their moral beliefs when exposed to safety and professionalism breaches or challenges. Due to our qualitative findings indicating that students change their interpretation of the problematic event based on their decision to act in a morally courageous way and that some are exposed to specific professionalism violations while others are not when designing the questionnaire, we decided to make the cases not explicit to specific types of professionalism breaches – e.g., not focused on talking above a patient’s head [1], but rather general the type of behavior e.g., “behaves immorally”. This decreases the personal interpretation if one behavior is acceptable by this individual; and also decreases the possibility of not answering the question if the individual student has never seen that specific behavior. Furthermore, to avoid “gray areas” in moral issues, we wrote the statements in a manner where there is no doubt whether there is a moral problem (“problematic situation”) [47], and thus the focus was only on one’s feeling of being capable of speaking up about their concern, i.e., act in a moral courage efficacy (see Table 1).

The instrument’s initial development consisted of 14 items addressing various populations, including senior MDs. The 14-item tool included questions regarding the willingness to recommend a second opinion or to convey one’s medical mistake to patients and their families. These actions are less relevant to MS. Thus, we extracted the questionnaire to a parsimonious instrument of 8 items.

The 8 items were divided into two dimensions: others and the self. This division is supported by the literature on moral courage that distinguishes between courage regarding others- vs. self-behavior. Hence, the questionnaire was designed to assess one’s perceived ability to act/speak up in these two dimensions: (a) situations of moral courage efficacy relating to others’ behavior (e.g., “*capable of telling a senior physician if I have detected a mistake s/he might have made*”); (b) situations of moral courage efficacy relating to self (e.g., “*capable of disclosing my mistakes to a senior physician*”). This two-dimension division is important and was absent in former measurements of moral courage. It was also replicated in another study we conducted among MS [49]. Furthermore, factor analysis with Oblimin rotation supported this two-factor structure (Table 1). All items had a high factor loading on the relevant factor (it should be mentioned that item 4 was loaded 0.59 on the relevant factor and 0.32 on the non-relevant factor).

All items are rated on a 5-point Likert scale (0 = to a small extent; 4 = to a very great extent) and are calculated by averaging the answers on the dimension, with higher scores representing higher moral courage efficacy. Internal reliability was α = 0.80 for the “others” dimension and α = 0.84 for the “self” dimension.

**Table 1:** Factor analysis with Oblimin rotation for moral courage efficacy instrument

Items	Factor 1	Factor 2
1. Capable of intervening when a physician behaves immorally toward a patient	**0.84**	-0.08
2. Capable of expressing my feelings of discomfort to a senior physician following his/her problematic behavior toward patients, or following problematic behavior toward patients by another physician in the department	**0.85**	-0.04
3. Capable of telling a senior physician if I have identified a concern about an error s/he might have made	**0.80**	0.10
4. Capable of telling a senior physician that I have been asked to perform a task that is against my moral principles	**0.59**	0.32
5. Capable of disclosing my mistakes to a senior physician	0.06	**0.77**
6. Capable of disclosing my lack of knowledge and of asking when in doubt	-0.03	**0.83**
7. Capable of saying that I lack the competence expected of me to perform a medical procedure on a patient	-0.01	**0.87**
8. Capable of inviting criticism and feedback in various situations	-0.03	**0.80**
*% of explained variance*	46.64	19.43

*Perceived stress*—This single-item questionnaire *(“How would you rate the level of stress you’ve been experiencing in the last few days?”*) evaluates MS’ perceived stress currently in their life on an 11-point Likert scale (0 = no stress; 10 = extreme stress), with higher scores representing higher perceived stress. It is based on a similar question evaluating MS’ perceived emotional stress [29]. Even though a multi-item measure might be more stable, previous studies indicated that using a single item is a practical, reliable alternative, with high construct validity in the context of felt/perceived stress, self-esteem, health status, etc [43, 50, 51].

*Academic burnout*—This 15-item instrument is a translated version [44] of the MBI-SS (MBI–Student Survey) [37], a common instrument used to measure burnout in the academic context, e.g. MS [52, 53]. It measures students’ feelings of burnout regarding their studies on three dimensions: (a) ‘exhaustion’ (5 items; e.g., “*Studying or attending a class is a real strain for me*”), (b) ‘cynicism’ (4 items; e.g., “*I doubt the significance of my studies*”), (c) lack of personal academic efficacy (‘reduced professional efficacy’) (6 items; “*I feel [un]stimulated when I achieve my study goals*”). Each item is rated on a 7-point Likert scale (0 = never; 6 = always) and is calculated by summing the answers on the dimension (after re-coding all professional efficacy items), with higher scores representing more frequent feelings of burnout. Internal reliability was α = 0.80 for ‘exhaustion’, α = 0.80 for ‘cynicism’, and α = 0.84 for ‘reduced professional efficacy’.

### Statistical analyses

IBM-SPSS (version 25) was used to analyze the data. Pearson’s correlations examined all possible bivariate associations between the study variables. PROCESS macro examined the mediation effects (via model#4). The significance of the mediation effects was examined by calculating 5,000 samples to estimate the 95% percentile bootstrap confidence intervals (CIs) of indirect effects of the predictor on the outcome through the mediator [54]. *T*-tests for independent samples examined differences between the study variables in the pre-clinic and clinic stages. The defined significance level was set generally to 5% (*p* < 0.05).

## Results

This study focused on understanding moral courage efficacy, i.e., MS’ perceived ability to speak up and act while exposed to others’ and their own wrongdoing. The sample’s frequencies demonstrate that only 10% of the MS reported that their moral courage efficacy toward the others was “very high to high,” and 54% reported this toward the self. Mean scores demonstrate that regarding the others, MS showed relatively low/moderate levels of moral courage and higher levels regarding the self. As for the variables tested to be associated with moral courage efficacy, MS showed relatively high perceived stress and low-to-moderate academic burnout (see Table 2 for the variables’ psychometric characteristics).

**Table 2:** Psychometric characteristics and correlations of the study variables

	1	2	3	4	5	6
1. Moral courage efficacy – others	--	0.44***	-0.11	-0.12	-0.08	-0.23***
2. Moral courage efficacy – self		--	-0.20**	-0.27***	-0.20**	-0.24***
3. Stress			--	0.60***	0.35***	0.16*
4. Burnout – exhaustion				--	0.67***	0.24***
5. Burnout – cynicism					--	0.43***
6. Burnout – academic efficacy						--
*M*	1.69	2.85	6.45	18.29	9.47	15.63
*SD*	0.88	0.84	2.53	6.74	6.41	6.30
*Range*	0–4	0–4	0–10	3–30	0–24	2–33
*α*	0.80	0.84	--	0.84	0.86	0.71

*p < 0.05; **p < 0.01; ***p < 0.001

*M* = mean.

*SD* = standard deviation.

*Range*-minimum–maximum.

*α* = Cronbach’s alpha.

Table 2 also shows the correlations among the study variables. According to Cohen’s (1988) [55] interpretation of the strength in bivariate associations (Pearson correlation), the effect size is low when r value varies around 0.1, medium when it is around 0.3, and large when it is more than 0.5. Hence, regarding the associations between the two dimensions of moral courage efficacy: we found a moderate positive correlation between the efficacy toward others and the efficacy toward the self. Regarding the associations among the three academic burnout dimensions: we found a strong positive correlation between ‘exhaustion’ and ‘cynicism,’ a weak positive correlation between ‘exhaustion’ and ‘reduced professional efficacy,’ and a moderate positive correlation between ‘cynicism,’ and ‘reduced professional efficacy.’

As for the associations concerning H1, Table 2 indicates that one academic burnout dimension, i.e., ‘reduced professional efficacy,’ had a weak negative correlation with moral courage efficacy toward the others, thus high burnout was associated with lower perceived moral courage efficacy toward others. Additionally, perceived stress and all three burnout dimensions had weak negative correlations with moral courage efficacy toward the self—partially supporting H1.

As for the associations concerning H2, Table 2 indicates that perceived stress had a strong positive correlation with ‘exhaustion,’ a moderate positive correlation with ‘cynicism,’ and a weak positive correlation with ‘reduced professional efficacy’—supporting H2.

**Fig. 1:**
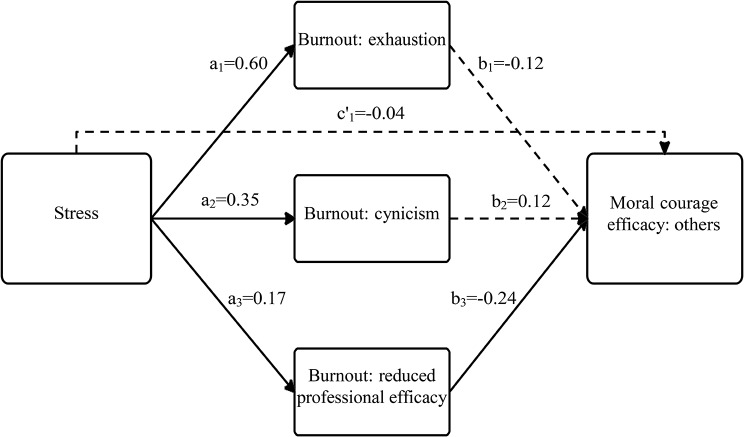
A model presenting the association between perceived stress and moral courage efficacy toward others, mediated by academic burnout. *Note* full arrows contain significant β coefficient values (fractured arrows mean nonsignificance

**Fig. 2:**
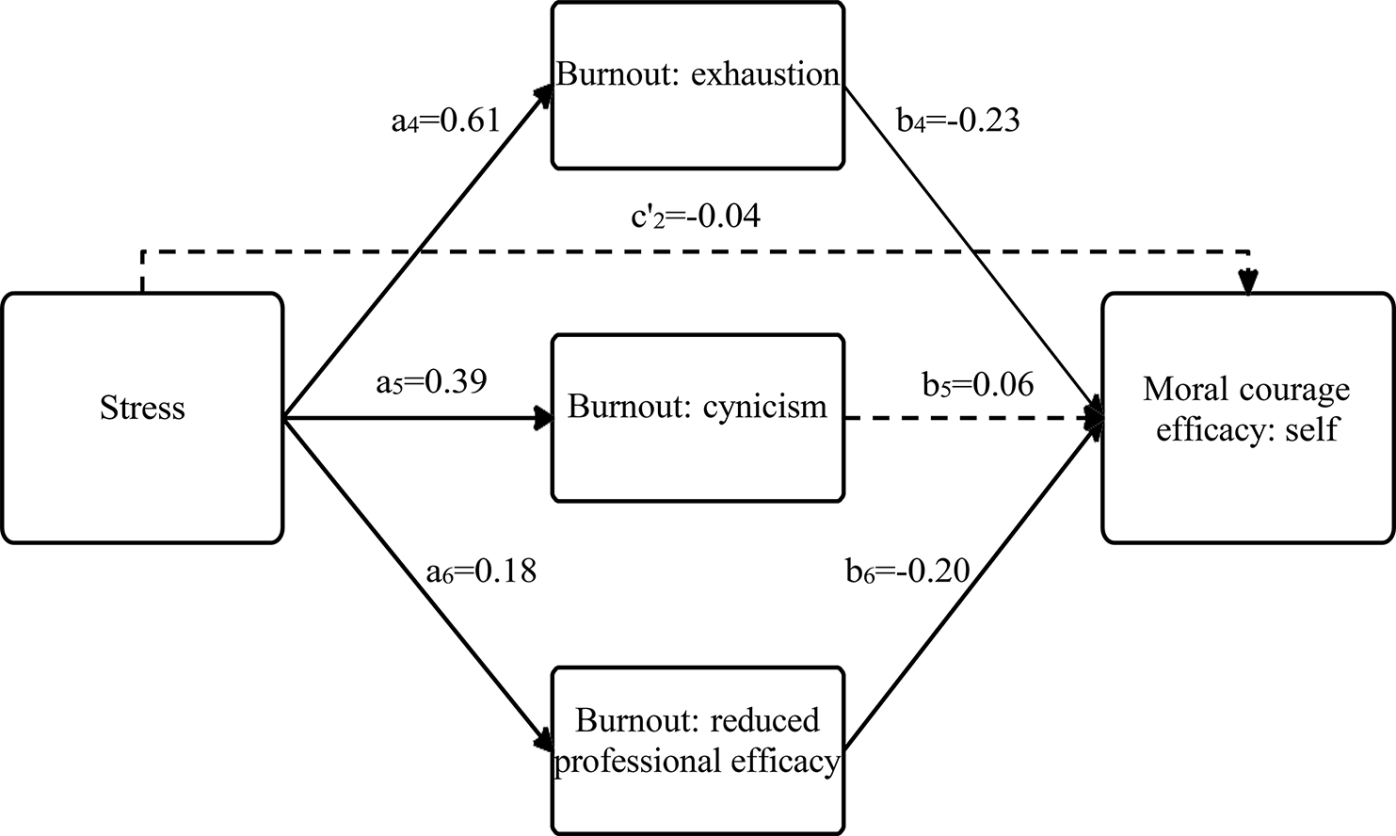
A model presenting the association between perceived stress and moral courage efficacy towards self, mediated by academic burnout. *Note* full arrows contain significant β coefficient values (fractured arrows mean non-significance

Based on these correlations, we conducted regression-based models to examine the unique and complex relationships among the study variable, including their various dimensions, while focusing on the examination of whether academic burnout mediates the association between perceived stress and moral courage efficacy (see Tables 3 and 4; and Figs. 1 and 2).

**Table 3:** Total, direct, and indirect effects of perceived stress on moral courage efficacy toward others through academic burnout

Predictor	B	SE	β	T		LLCI, ULCI	
	Mediator – Burnout: exhaustion						
Stress (path a_1_)	1.60	0.14	0.60	11.17		1.31, 1.88	
	Mediator – Burnout: cynicism						
Stress (path a_2_)	0.88	0.16	0.35	5.58		0.57, 1.19	
	Mediator – Burnout: reduced professional efficacy						
Stress (path a_3_)	0.43	0.16	0.17		2.63		0.11, 0.75
	Dependent variable – Moral courage: others						
Burnout: exhaustion (path b_1_)	-0.02	0.01	-0.12	-1.12		-0.04, 0.01	
Burnout: cynicism (path b_2_)	0.02	0.01	0.12	1.29		-0.01, 0.04	
Burnout: reduced professional efficacy (path b_3_)	-0.03	0.01	-0.24	-3.34		-0.05, -0.01	
Stress (path c_1_ = total effect)	-0.04	0.02	-0.11	-1.66		-0.08, 0.01	
Stress (path c’_1_=direct effect)	-0.01	0.03	-0.04	-0.51		-0.07, 0.04	
Stress -> Burnout: exhaustion (path a_1_b_1_ = indirect effect)	-0.02	0.02	-0.07	--		-0.06, 0.01	
Stress -> Burnout: cynicism (path a_2_b_2_= indirect effect)	0.01	0.01	0.04	--		-0.01, 0.04	
Stress -> Burnout: reduced professional efficacy (path a_3_b_3_ = indirect effect)	-0.01	0.01	-0.04	--		-0.03, -0.00	

B = unstandardized beta.

*SE* = standard error for the unstandardized beta.

β = standardized beta.

*T* = *t*-test statistic.

*LLCI–ULCI* = lower limit of the confidence interval–upper limit of the confidence interval.

### Focusing on moral courage efficacy toward others

Table 3 and Fig. 1 indicate that perceived stress was positively associated with all three academic burnout dimensions: ‘exhaustion’ (path a_1_), ‘cynicism’ (path a_2_), and ‘reduced professional efficacy’ (path a_3_). These paths support H2. In turn, ‘reduced professional efficacy’ was negatively associated with moral courage efficacy toward the others (path b_3_), supporting H1. The CIs of the indirect effect (paths a_3_b_3_) did not contain zero; therefore, perceived stress had a significant indirect effect on moral courage efficacy toward the others, through the burnout dimension ‘reduced professional efficacy.’ This path supports H3.

**Table 4:** Total, direct, and indirect effects of perceived stress on moral courage efficacy toward the self through academic burnout

Predictor	B	SE	β	T		LLCI, ULCI	
	Mediator – Burnout: exhaustion						
Stress (path a_4_)	1.66	0.15	0.61	11.33		1.37, 1.95	
	Mediator – Burnout: cynicism						
Stress (path a_5_)	1.00	0.16	0.39	6.29		0.69, 1.31	
	Mediator – Burnout: reduced professional efficacy						
Stress (path a_6_)	0.47	0.17	0.18		2.78		0.14, 0.80
	Dependent variable – Moral courage: self						
Burnout: exhaustion (path b_4_)	-0.03	0.01	-0.23	-2.29		-0.05, -0.00	
Burnout: cynicism (path b_5_)	0.01	0.01	0.06	0.68		-0.02, 0.03	
Burnout: reduced professional efficacy (path b_6_)	-0.03	0.01	-0.20	-2.84		-0.05, -0.01	
Stress (path c_2_ = total effect)	-0.07	0.02	-0.20	-2.96		-0.11, -0.02	
Stress (path c’_2_=direct effect)	-0.01	0.03	-0.04	-0.51		-0.07, 0.04	
Stress -> Burnout: exhaustion (path a_4_b_4_ = indirect effect)	-0.05	0.02	-0.14	--		-0.09, -0.01	
Stress -> Burnout: cynicism (path a_5_b_5_ = indirect effect)	0.01	0.01	0.03	--		-0.02, 0.04	
Stress -> Burnout: reduced professional efficacy (path a_6_b_6_ = indirect effect)	-0.01	0.01	-0.04	--		-0.03, -0.00	

B = unstandardized beta.

*SE* = standard error for the unstandardized beta.

β = standardized beta.

*T* = *t*-test statistic.

*LLCI–ULCI* = lower limit of the confidence interval–upper limit of the confidence interval.

### Focusing on moral courage efficacy toward the self

Table 4 and Fig. 2 also indicate that perceived stress was positively associated with all three academic burnout dimensions: ‘exhaustion’ (path a_4_), ‘cynicism’ (path a_5_), and ‘reduced professional efficacy’ (path a_6_). These paths support H2. In turn, ‘exhaustion’ and ‘reduced professional efficacy’ were negatively associated with moral courage efficacy toward the self (paths b_4_, b_6_ respectively). These paths support H1 The CIs of the indirect effects (paths a_4_b_4_, a_6_b_6_) did not contain zero; therefore, perceived stress had a significant indirect effect on moral courage efficacy toward the self, through the burnout dimensions ‘exhaustion’ and ‘reduced professional efficacy.’ These paths support H3. It should be noted that in this analysis, the initially significant association between perceived stress and moral courage efficacy toward the self (path c_2,_ representing H1) became insignificant in the existence of academic burnout dimensions (path c’_2_). These results demonstrate complete mediation and also support H3.

In addition to examining the complex relationships between stress, academic burnout, and moral courage efficacy among MS, we tested the differences between MS in the pre-clinical and clinical school stages in all study variables. The results indicate non-significant differences in moral courage efficacy. However, medical-school-stage differences were found in stress [t(197.4)=-4.36, *p* < 0.001] and in one academic burnout dimension [t(233)=-2.40, *p* < 0.01]. In that way, MS at the clinical stage reported higher levels of perceived stress (*M* = 7.32; *SD* = 2.17) and exhaustion (*M* = 19.67; *SD* = 6.58) than MS in the pre-clinical stage (*M* = 5.94; *SD* = 2.59 and *M* = 17.48; *SD* = 6.78, respectively).

## Discussion

This study examined the associations between perceived stress, academic burnout, and moral courage efficacy to understand MS’ perceived ability to speak up and act while exposed to others’ and their own wrongdoing. The findings show that one dimension of burnout, that of ‘reduced professional efficacy,’ mediated the associations between perceived stress and moral courage efficacy toward both others and self. ‘Exhaustion’ mediated the association between perceived stress and moral courage efficacy only toward the self.

Before discussing the meanings of the associations, this study was an opportunity to explore moral courage efficacy occurrence. The findings indicated fairly low/moderate mean scores of perceived ability to speak up and act while confronted with others’ wrongdoing and moderate/high scores of perceived ability while confronted with one’s own wrongdoing. This implies that students do not feel capable enough to share their concerns regarding others’ possible errors and feel more able, but still not enough, to share their own flaws and needs for guidance. These findings require attention, from both patient safety and learning perspectives.

Regarding patient safety, feeling unable to act while confronted with others or self- wrongdoing means that some errors may occur and not be addressed. This is in line with former findings that showed that less than 50% of MS would actually approach someone performing an unsafe behavior [12], or report an error they had observed [15]. These numbers are likely to improve in postgraduates as studies showed that between 64 and 79% of interns and residents reported they would likely speak up to an attending when exposed to a safety threat [56, 57]. 

Regarding learning, our MS’ scores must improve for various reasons. First, moderate scores may indicate a psychologically unsafe learning environment, which prevents or discourages sharing uncertainties, especially about others’ behavior, and creates difficulty for students to share their own concerns, limitations, mistakes, and hesitations when feeling incapable or unqualified for a task [58]. Second, limited sharing of errors may be problematic because by not disclosing their error, students miss the chance to learn from it; [59] they lose the opportunity for reflective guidance to explore what worked well, what did not, and how to improve [59, 60]. Third, if they do not discuss others’ errors or their own, they may deny themselves the necessary support to learn the all-important skills of how to deal with the emotional turmoil and challenges of errors, and how to share the error with a patient or family member [61]. Furthermore, if MS feel incapable of sharing their concern about a senior’s possible mistake, they miss other learning opportunities—e.g., the senior’s reasoning and clinical judgment may show that a mistake was not made. In this case, the student would miss being shown why they were wrong and what they did do well. Thus, identifying what can enhance moral courage efficacy and practice is needed. The fact that there are no significant differences between pre-clinical and clinical years students in their perceived ability to apply moral courage, may indicate that there is a cultural barrier in perceiving the idea of sharing weakness or of revealing others’ mistakes as unacceptable. Thus, the socialization, in the medical school environment, both in pre-clinical and clinical years, perhaps lacks the encouragement to speak up and provision of safe space.

This study examined the associations between perceived stress, academic burnout, and moral courage efficacy among MS. The findings indicate that, like earlier studies, stress is not directly connected to speaking up [62] or moral courage. It rather contributes to it indirectly, through the impact of burnout. Beyond the well-established role of stress in explaining burnout [63, 64], we identified a negative consequence of burnout—hindering moral courage efficacy. This may help explain the path in which previous studies found burnout to impair MS’ quality of life, how it leads to dropout, and to more medical errors [65]. When individuals experience the burnout dimension of ‘reduced professional efficacy,’ they may feel less confident and fit, leading them to feel more disempowered to take the risk (required in courage) and share their concerns and hesitations about others’ mistakes and their own challenges. This fits earlier studies indicating that being a young professional experiencing “lack of knowledge” or “unfamiliarity” with clinical subtleties is a barrier to moral courage [11]. This may have various negative implications, of limited moral courage efficacy, as seen here, as well as paying less attention and not fully addressing their learning needs, leading to a vicious cycle of “feeding” the misfit feeling, potentially increasing their moral distress. Furthermore, those who feel they know less and, therefore, need more support to fill the gap in knowledge and skills, are less inclined to ask for help.

Beside the negative associations between ‘reduced professional efficacy’ and both dimensions of moral courage efficacy (toward others and the self), another dimension of academic burnout—‘exhaustion’—was negatively associated with moral courage efficacy toward the self. This is worrying because when learners are exhausted, their attention is reduced and they are at greater risk of error, as proven in an earlier study [65]. The current study adds to this information another worry, showing that MS are less willing to share their hesitations about themselves or the mistakes they already made, thus perhaps not preventing the error or fixing it. MS might create an unspoken contract with senior physicians about not exposing each other’s mistakes, with various possible negative implications. Some MS’ tendency to defend physicians’ mistakes was identified elsewhere [66]. 

The findings concerning medical-school-stage differences demonstrated that MS in the clinical stage had higher perceived stress and exhaustion levels than MS in the pre-clinical stage. These results support previous studies indicating stress, academic burnout, and more challenging characteristics among more senior students, including a decline in ideals, altruistic attitudes, and empathy during medical school studies; or more exhaustion, cynicism, and higher levels of detached emotions and depression through the years of medical school [67–69]. These higher levels of stress and exhaustion, can be explained by the senior students’ exposure to the rounds in the hospitals, which requires ongoing learning, more pressure, and a sense of overload in their academic life.

### Limitations and future studies

Despite the importance of the findings, the study has several limitations. First, the participants were from one university, and recruited via convenience sampling, including only MS who voluntarily completed the questionnaires, undermining generalizability. To address this limitation, future research should aim to include a more diverse and representative sample of medical students from multiple universities and geographical regions. This would enhance the external validity and applicability of the findings across different educational and cultural contexts. Second, future studies are recommended to follow up on medical students’ stress, academic burnout, and moral courage efficacy over time. Exploring the development of professional efficacy and the barriers to exposing one’s and others’ weaknesses and flaws within the medical environment can help improve the medical culture into a safer space. Third, an intriguing avenue for future research is the exploration of the construct of ‘moral courage efficacy’ within different cohorts of healthcare students throughout their undergraduate and postgraduate years to learn about their moral courage efficacy development as well as and to verify the association between the findings from this newly developed scale and actual moral courage behavior. Additionally, experimental designs, such as interventions to reduce stress and burnout among medical students, could be employed to observe the impact on moral courage efficacy.

### Conclusions and implications

This study is a first step in understanding moral courage efficacy and what contributes to it. The study emphasizes the importance of promoting MS’ well-being—in terms of stress and burnout—to enhance their moral courage efficacy. The findings show that the ‘reduced professional efficacy’ mediated the association between perceived stress and moral courage efficacy, toward both the others and self. This has potential implications for safety, learning, and well-being. To encourage MS to develop moral courage efficacy that will potentially increase their morally courageous behavior, we must find ways to reduce their stress and burnout levels. As the learning and work environments are a major cause of burnout [38], it would be helpful to focus on creating safe spaces where they can share others- and self-related concerns [70]. The first step is a learning environment promoting students’ overall health and well-being [71]. Useful additions are processes that support MS while dealing with education- and training-related stresses, improving their academic-professional efficacy, and constructively helping them handle challenging situations through empathic feedback [70]. This can lead them to a stronger belief in their ability to share safety and professionalism issues, thus enhancing their learning and patient care.

## Data Availability

No datasets were generated or analysed during the current study.

## Declarations

### Previous presentations

Study findings were presented at the Academy for Professionalism in Healthcare Conference, June 2nd, 2022, virtual; and the 7th International Conference on Public Health, August 8th, 2021, virtual.
